# Aberrant Proliferation in CXCR7+ Endothelial Cells via Degradation of the Retinoblastoma Protein

**DOI:** 10.1371/journal.pone.0069828

**Published:** 2013-07-23

**Authors:** Jennifer E. Totonchy, Jessica M. Osborn, Sara Botto, Lisa Clepper, Ashlee V. Moses

**Affiliations:** Vaccine and Gene Therapy Institute, Oregon Health and Science University, Portland, Oregon, United States of America; Medical College of Wisconsin, United States of America

## Abstract

Angiogenesis is a critical factor in the growth and dissemination of solid tumors. Indeed, tumor vasculature is abnormal and contributes to the development and spread of malignancies by creating a hostile microenvironment. The alternative SDF-1/CXCL12 receptor, CXCR7, is frequently and specifically expressed in tumor-associated vessels. In this study, we examine the role of endothelium-expressed CXCR7 in tumor vascular dysfunction by specifically examining the contribution of CXCR7 to endothelial cell (EC) proliferation. We demonstrate that CXCR7 expression is sufficient to drive post-confluent growth in EC cultures. Further, we provide a novel mechanism for CXCR7-mediated proliferation via proteasomal degradation of the tumor suppressor protein Rb. These findings identify a heretofore unappreciated role for CXCR7 in vascular dysfunction and confirm this receptor as a plausible target for anti-tumor therapy.

## Introduction

Angiogenesis is the process by which new vessels form from existing vascular networks in both the blood and lymphatic circulatory systems. This highly regulated process is critical for wound healing and tissue regeneration but is co-opted in a variety of pathogenic processes including angioproliferative diseases and the growth of aberrant vasculature into tumors [1]. Endothelial cells (EC) line all vessels and are key players in the angiogenic process. In normal vessels, EC are long-lived, quiescent cells that are highly dependent upon cell-cell and cell-substrate adhesion for their survival and function. Angiogenesis requires both EC migration into an angiogenic niche and EC proliferation in order to form new vascular structures [2].

The vasculature that forms in the tumor microenvironment is structurally and functionally abnormal compared to vessels formed during normal wound healing. This vascular dysfunction is a direct result of abnormalities in EC function and vessels formed by this pathological process do not allow correct circulation within the tumor tissue. The result is a hostile tumor microenvironment characterized by abnormally high interstitial pressure, low pH, poor oxygenation and poor immune surveillance. Tumor vascular dysfunction exacerbates the development and spread of cancer by selecting for tumor cells that can survive and proliferate under these adverse conditions, thereby enhancing malignancy and driving the development of metastases [3].

Chemokines and their receptors are important players in pathological angiogenesis [4] as well as the migration and invasion of tumor cells [5], [6]. The chemokine SDF-1/CXCL12 and its canonical receptor CXCR4 are among the most highly studied chemokine/receptor pairs in cancer biology [7], [8]. A second receptor for SDF-1/CXCL12 was recently discovered and designated CXCR7 [9]. Since its discovery as an alternative receptor for SDF-1/CXCL12, a number of studies have explored the expression of CXCR7 in tumors. CXCR7 is sporadically expressed by tumor cells in renal [10], breast [11], [12], lung [12], liver [13], prostate [14] and central nervous system [15] cancers and the implications of CXCR7 expression for malignant progression are currently an area of intense investigation. EC express very low levels of CXCR7 under normal physiological conditions *in vivo* [12] and normal culture conditions *in vitro* [9], but CXCR7 is induced in activated EC [9] suggesting that CXCR7 may participate in the response of EC to physiological stress. Interestingly, several studies have shown that tumor-associated EC display aberrant CXCR7 expression in a variety of cancer models [11], [16], [17]. Indeed, one such study by Miao et. al. demonstrated profound and highly specific expression of CXCR7 in vessels associated with malignancies of the breast, lung, liver, ovary, kidney and bladder, leading the authors to postulate that CXCR7 might be a specific marker of tumor endothelium [12]. Despite the fact that CXCR7 expression is extensively and specifically observed on tumor-associated vasculature in both primary tumors and metastases, the functional consequences of CXCR7 expression in EC remain poorly understood.

Although CXCR7 expression has been linked to cellular proliferation both in cancer models [9], [12], [13], [17], [18], and EC cultures [19], the current literature fails to establish the mechanisms by which CXCR7 influences cell division. In this study, we investigate CXCR7-mediated proliferation in adult primary lymphatic EC. We demonstrate that CXCR7+ EC cultures display post-confluent proliferation, the extent of which corresponds to their level of CXCR7 expression. We discover via a protein microarray screen that CXCR7 influences the expression of multiple proteins involved in cell cycle control, including the tumor suppressor protein retinoblastoma (Rb). We verify that that CXCR7+ EC cultures have reduced levels of Rb and that Rb expression can be rescued by inhibiting the proteasome. We use ligand neutralization, pharmacological receptor targeting and receptor mutagenesis to establish that the loss of Rb in CXCR7+ EC requires CXCR7 ligands and that rescue of Rb expression is associated with decreased proliferation. These results identify CXCR7 as a mediator of aberrant proliferation in EC and establish CXCR7 as a potential therapeutic target for pathological angioproliferation in cancer.

## Materials and Methods

### Cell Lines and Reagents

Primary Human Adult Lymphatic and Blood Vascular Endothelial Cells (pLEC and pBEC, respectively) were commercially obtained (Lonza) and maintained in EGM-2 medium (Lonza #CC-3024) containing bullet kit growth supplements (Lonza #CC-3124), 10% fetal bovine serum (FBS, Hyclone SH30396.03) and Penicillin/Streptomycin/L-Glutamine (PSG, Hyclone SV30082.01). All primary EC cultures were used between passage 4 and 10. MouseIgG1-anti-CXCR7 antibody (11G8) was provided by Chemocentryx, Inc (Mountain View, CA), MouseIgG2b-anti-CXCR7 antibody (clone 8F11, 331102), AlexaFluor647-conjugated mouseIgG1-anti-CXCR3 antibody (334903) and Mouse-IgG1 isotype control (401402) were from Biolegend. Mouse-IgG2b (556654) and Pacific Blue Mouse–IgG2a (558118) isotype controls were from BD Pharmingen. MouseIgG2a-anti-CD31/PECAM antibody for IFA was from Thermo/Fisher (MA3100). Rabbit-anti-HA (sc-805), Rabbit-IgG Isotype control (sc-2027) and HRP-conjugated anti-mouse (sc-2306) anti-rabbit (sc-2305) were from Santa Cruz Biotechnology. Rb antibody was from Cell Signaling (9309) and GAPDH antibody was from Abcam (ab8245). Anti-mouseIgG2a-AlexaFluor488 (A21131), anti-mouseIgG1-AlexaFluor594 (A21125), anti-rabbit-AlexaFluor488 (A110088) and Streptavidin-Pacific Blue (S11222) were from Invitrogen. HA-HRP direct conjugate was from Roche (12013819011). Recombinant human SDF-1/CXCL12 (350-NS-050) and neutralizing antibodies against SDF-1/CXCL12 (AF-310-NA) and ITAC/CXCL11 (AF260) and biotinylated MouseIgG2a-anti-CXCR4 antibody (clone 12G5, FAB170B) were from R&D Systems. The CXCR7 small molecule antagonist CCX733 was a generous gift from Chemocentryx, Inc. Propidium Iodide was from Sigma (P4170).

### CXCR7 Adenovirus Vectors

CXCR7-expressing vectors with an N-terminal HA tag were constructed by subcloning the DNA fragment into pAdTet7. This vector contains the tet-responsive enhancer within a minimal CMV promoter followed by the SV40 late poly(A) cassette, adenovirus E1A, and a single loxP site to increase recombination frequency. Recombinant adenoviruses were produced by co-transfection of 293 cells expressing the Cre recombinase with adenovirus DNA (Ad5-ψ5) that contains an E1A/E3-deleted adenovirus genome and pAdtet7-HA-CXCR7 or pAdtet7-HA-CXCR7-Δ5. Recombinant adenoviruses were expanded on 293-Cre cells, and the bulk stocks were titered on 293 cells by limiting dilution. Expression was driven by co-infection with Ad-Trans expressing the Tet-off transactivator.

### Infection of EC with Adenovirus Vectors

Adenovirus infections were performed on confluent EC cultures in endothelial SFM medium (endoSFM, Gibco 11111) supplemented with 1% FBS, PSG and 37.5 mg/ml endothelial cell growth supplement (ECGS, BD Biosystems 356006). Where appropriate, infection medium was replaced with medium containing drugs or neutralizing antibodies at 6 hours post-infection.

*Indirect Immunofluorescence and Image Analysis -* EC were plated on collagen-coated coverslips (BD Biosystems 354089) and infected with either Trans at MOI 100 only or Trans at MOI 100 and CXCR7 at MOI 100. At 20 hours post-infection, cells were washed once with phosphate buffered saline containing calcium and magnesium (PBS+) and fixed in PBS+ containing 2% paraformaldehyde (PFA). Coverslips were blocked for 15 min at room temperature (RT) in PBS+0.2% saponin+2% normal goat serum (NGS). All further incubations were performed in PBS+0.2% saponin+0.2% NGS. Primary antibodies were diluted 1∶200 and applied for 30 minutes at RT. Secondary antibodies and 4′,6-diamidino-2-phenylindole (DAPI) were diluted 1∶1000 and applied for 30 minutes at RT. Coverslips were washed and mounted on glass slides with FluoromountG (Southern Biotech, 0100-01). For the barrier formation studies, cultures were trypsinized at 20 hours post-infection, counted and 2(10)^5^ cells were replated in duplicate into 8-well Permanox chamber slides (NUNC 1177445) coated with 1% gelatin, allowed to form a new monolayer for a further 20 hours then fixed in PBS+ containing 2% PFA and 1% TritonX-100 for 15 minutes at RT. Coverslips were then post-fixed for a further 5 minutes at RT in PBS+ containing 2% PFA only. Coverslips were blocked in PBS+ with 1% TritonX-100 and 2% NGS for 15 minutes at RT. All further incubations were performed in PBS+ with 1% TritonX-100 and 0.2% NGS (Tx Wash). Antibody concentrations were the same as above. Image acquisition was on a Deltavision real-time deconvolution (DVRT) microscope (Applied Precision) using a Photometrics CoolSNAP HQ camera. Image analysis was performed using Softworx (Applied Precision). Unless otherwise indicated, z-stacks with a 0.2 µm z-step size were taken at 60X magnification. Stacks were subjected to deconvolution analysis and 2–3 section projections were made superimposing representative z-planes to generate the final image.

### Flow Cytometry

Cells were dissociated with Cellstripper (Cellgro, 25-056-CI) and resuspended in cold PBS+ containing 2% NGS and 0.1% sodium azide (NaN3) (Surface Block) for 15 minutes on ice. Cells were then incubated for 15 minutes on ice with Rabbit anti-HA antibody diluted 1∶100 in cold PBS+ containing 0.2% NGS and 0.1% NaN3 (Surface Wash) followed by 15 minutes on ice with anti-rabbit Alexa488 secondary antibody at 1∶1000 dilution and 1 µM propidium iodide (PI) in 100 µl Surface Wash. Analysis was on a BD LSR2 flow cytometer. Live, non-necrotic cells were gated based on scatter characteristics and negative PI staining. Compensation settings were determined empirically for each experiment on single color controls using BD CompBeads for mouse antibodies (552843) and Flow cytometry protein A beads for rabbit antibodies (Bangs Laboratories 553) and unstained cells for PI.

### CyQuant Proliferation Assay

EC in 96-well plates were infected as above with Trans and CXCR7 at indicated doses. When applicable, drugs or inhibitors were added at 6 hours post-infection. At 20 hours post-infection cells were lysed in buffer containing CyQuant proliferation dye. Lysates were transferred to white-walled 96-well plates (Costar 3903) and fluorescence was read on a Molecular Devices Flexstation Plate Reader. All conditions were performed in at least 6 biological replicates per experiment. Individual experiments were normalized to the average of negative control cultures and at least two experimental replicates were averaged to achieve statistical power. P-values were generated by Student’s T-test using Microsoft Excel.

### Antibody Microarray

pLEC were infected in duplicate with either Trans only at MOI 100 or Trans MOI 100+ CXCR7 at MOI 50 in EBM-2+ Bullet Kit Supplements +1% FBS. At 18 hours post-infection cells were starved in serum free M199 for 1 hour then stimulated for 10 minutes at 37°C with 50 ng/ml rhSDF-1/CXCL12. A subset of cells were used to verify CXCR7 expression by flow cytometry (FACS) as described above and duplicate samples were pooled. Total cellular protein was extracted, biotinylated and hybridized to PEX100 microarray according to the manufacturer’s instructions (Full Moon BioSystems, Sunnyvale, CA). Completed slides were dried and scanned using a ScanArray Lite (Packard Bioscience) scanner. Optical density of spots and background subtraction was performed on the scanned images using TotalLab software. Optical density ratios and sorting were performed in Microsoft Excel.

### Western Blot Analysis

Cells were lysed in 200 µl modified radioimmunoprecipitation (RIPA) buffer (10 mM Tris pH 7.4, 150 mM NaCl, 1 mM EDTA, 1%Triton-X-100, 1% Sodium Deoxycholate, 0.1% SDS) containing protease inhibitors (Roche 11836145001) for 10 minutes on ice. Lysates were freeze-thawed once at −20°C and protein was quantified by BCA protein assay (Pierce, 23225). 0.5 µg of total lysate was boiled for 5 minutes in 1X LDS protein sample buffer containing reducing agent (Invitrogen, NP0009), loaded on NuPAGE 4–12% Bis-Tris gradient protein gels (Invitrogen, NP0335) and run in 1X NuPAGE MOPS buffer (Invitrogen, NP0001). Protein was transferred to Immobilon-P blotting membrane (Millipore, IPVH00010) and membranes were blocked by drying overnight at RT. Membranes were rehydrated in 2% ECL advance blocking buffer (Amersham, CPK1075) with 0.2% Tween-20 (Block) for 15 minutes. All subsequent incubations were performed in Block. Rb primary antibody was at 1∶2000 dilution and GAPDH primary antibody was at 1∶50,000 dilution 1 hour at RT. HRP-conjugated secondary antibodies were diluted 1∶40,000 and incubated for 20 minutes at RT. Blots were developed using ECL Advance (Amersham, RPN2135) chemiluminescent reagent and autoradiography. For all experiments, adenovirus transduction was verified by analysis of 0.2 µg of unboiled total protein blotted as above with a direct conjugate anti-HA-HRP. Densitometry analysis was performed on scanned western blot results using TotalLab software.

### Quantitative RT-PCR Analysis

Extraction of total RNA was performed on cell pellets using miRNeasy Mini Kit (Qiagen), DNase I treatment (Qiagen) was employed to degrade DNA from the RNA samples. Total cDNA was produced by reverse transcribing 200 ng of RNA, using SuperScript III First-Strand Synthesis System (Invitrogen). The primers employed for the amplification of the target genes by qPCR were the following: GAPDHF 5′-GAAGGTGAAGGTCGGAGT-3′, GAPDHR 5′-GAAGATGGTGATGGGATTTC-3′; SDF-1 F 5′-CTGTCACTGGCGACACGTAG -3′, SDF-1 R 5′-TCCCATCCCACAGAGAGAAG-3′; CXCR7 F 5′-CTGCGTCCAACAATGAGACCT-3′, CXCR7 R 5′-CCGATCAGCCACTCCTTGA-3′; ITAC-F 5′-GTTCAAGGCTTCCCCATGTTC-3′, ITAC-R 5′-CCACTTTCACTGCTTTTACCCC-3′. To perform the qPCR, 10 ng of cDNA (5 ul of sample) were loaded on a 96-well optical plate together with 20 ul of Power SYBR Green PCR Master Mix (ABI) containing the appropriate set of primers, for a final volume of 25 ul. Evaluation of primer and sample concentrations has been optimized for all the targets. Amplification of the samples was performed on Real Time PCR 7500 System (ABI), according to the manufacturer’s instructions. Normalized relative gene expression levels were calculated by applying the ΔΔCT method, which compares the expression of target genes in treated samples (Adenovirus-infected cells) to the one in control samples (mock-infected cells). For the purpose of this quantification, GAPDH was utilized as internal control and the results were represented as change in the expression level of each particular gene (fold change).

### Quantibody Antibody Array for Supernatant Cytokine Analysis

pLEC in 3.5 cm dishes were infected as above with Trans at MOI 200 or Trans+CXCR7 at MOI 100 and incubated for 20 hours at 37°C. At 20 hours post-infection, media was replaced with 0.5 ml serum free M199 and incubated for 4 hours at 37°C. After incubation supernatants were frozen in four 250 µl aliquots. Cells were harvested and analyzed for CXCR7 transduction by flow cytometry. Duplicate samples were collected from two independent experiments. Quantibody arrays were stained according to manufacturers instructions. Briefly, slides were blocked in sample diluent provided for 30 minutes at RT, cytokine standard curves were prepared and samples and standards were hybridized to the array at RT for 2 hours followed by 5X 5 minute washes. Detection antibody was hybridized to slides for 2 hours at RT followed by 5X 5 minute washes. Cy3 streptavidin was added for 1 hour at RT, protected from light followed by 5X 5 minute washes. The array slide was disassembled and air dried. Scanning and analysis service was performed by Ray Biotech.

## Results

### Adenovirus Expression System for CXCR7 in EC

**Figure 1:**
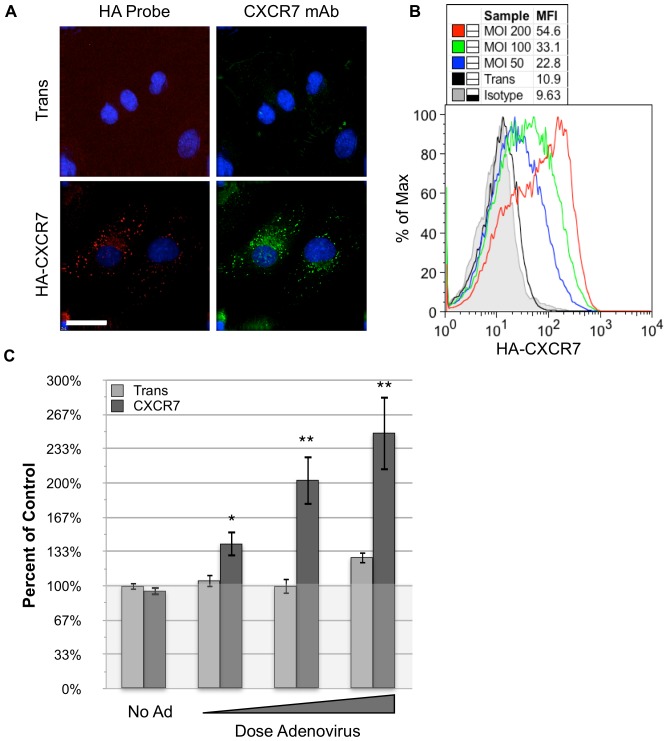
Adenovirus-transduced CXCR7+ EC display dose-dependent overproliferation. (A) pLEC were infected with Trans or Trans+CXCR7 at MOI 100. At 20 hours post-infection coverslips were fixed and stained for the HA-tag (red), CXCR7 protein via 11G8 monoclonal antibody (green) and DAPI. Images were taken at 60x magnification. White size bar indicates 30 µm. (B) pLEC were infected with Trans at MOI 50 or Trans+CXCR7 at indicated doses. At 20 hours post-infection cells were stained live for the HA-tag and analyzed by flow cytometry. Necrotic cells were excluded from the analysis via propidium iodide staining and scatter characteristics. Positive staining was compared to appropriate isotype control (grey) and mean fluorescence intensity values were calculated for HA fluorescence (MFI) (C) Confluent pLEC cultures were infected with increasing doses of Trans only or Trans+increasing doses of CXCR7. At 20 hours post-infection, cells were lysed in the presence of Cyquant. Fluorescence values are normalized to uninfected controls. n = 24 from three independent experiments. * *P* = 0.01, ** *P*<0.003.

In order to effectively study the ramifications of aberrant CXCR7 expression in an EC culture system, we constructed a Tet-inducible CXCR7-expressing adenovirus vector containing the complete CXCR7 sequence fused to an N-terminal HA-epitope tag. In this system, we achieve expression of CXCR7 via co-infection of EC with CXCR7 adenovirus and a second adenovirus vector expressing the tet-transactivator (hereafter referred to as Trans). In all experiments, cells transduced with Trans alone are used to control for nonspecific effects of adenovirus infection. Indirect immunofluorescence (IFA) analysis of HA-CXCR7-transduced EC demonstrate comparable staining with either an HA probe or the CXCR7 monoclonal antibody 11G8 (Figure 1A). Interestingly, this IFA analysis reveals a punctate intracellular staining pattern consistent with CXCR7 being primarily located in the secretory pathway and endosomes. In order to determine whether adenovirus-expressed CXCR7 is also displayed at the cell surface, we performed FACS analysis of cultures transduced with increasing doses of CXCR7, gating stringently on intact cells via propidium iodide exclusion. These experiments revealed that CXCR7 is expressed on the surface of adenovirus-transduced EC and that surface levels correlate with adenovirus dose (Figure 1B). We chose to do our experiments in primary human lymphatic EC (pLEC) because this lineage displays more robust growth in static culture compared to primary blood vascular lineage EC (pBEC) and has uniformly low levels of baseline CXCR7 expression compared to primary umbilical vein EC, in which we observe sporadic induction of CXCR7 in single cells (data not shown). However, we have verified key findings of the current study in pBEC (Figure S1) in order to verify that these phenotypes are not specific to the lymphatic lineage.

### CXCR7+ EC are Hyperproliferative

In order to determine whether CXCR7 mediates aberrant proliferation in EC cultures, we generated replication-quiescent pLEC cultures by growing cells to confluence and maintaining them at confluence for two days prior to initiating experiments. Quiescent, contact-inhibited EC were then transduced with increasing multiplicity of infection (MOI) of Trans only (0, 50, 100 or 200) or Trans at MOI 50 with increasing doses (MOI 0, 100, 200 or 300) of CXCR7 adenovirus. At 20 hours post-infection wells were lysed in the presence of CyQuant. We observed that cultures transduced with CXCR7 displayed a dose-dependent increase in overall cell numbers compared to uninfected or Trans-only controls. CXCR7-mediated overproliferation was statistically significant at all doses, while increasing doses of Trans alone did not result in significantly higher cell numbers compared to uninfected controls (Figure 1C). Because these cultures were quiescent prior to transduction, the proliferation observed in these experiments represents significant, aberrant post-confluent growth of EC as a result of CXCR7 expression. Similar results were obtained in pBEC cultures (Figure S1A).

### CXCR7+ EC are not Contact Inhibited and Form Abnormal Monolayers

**Figure 2:**
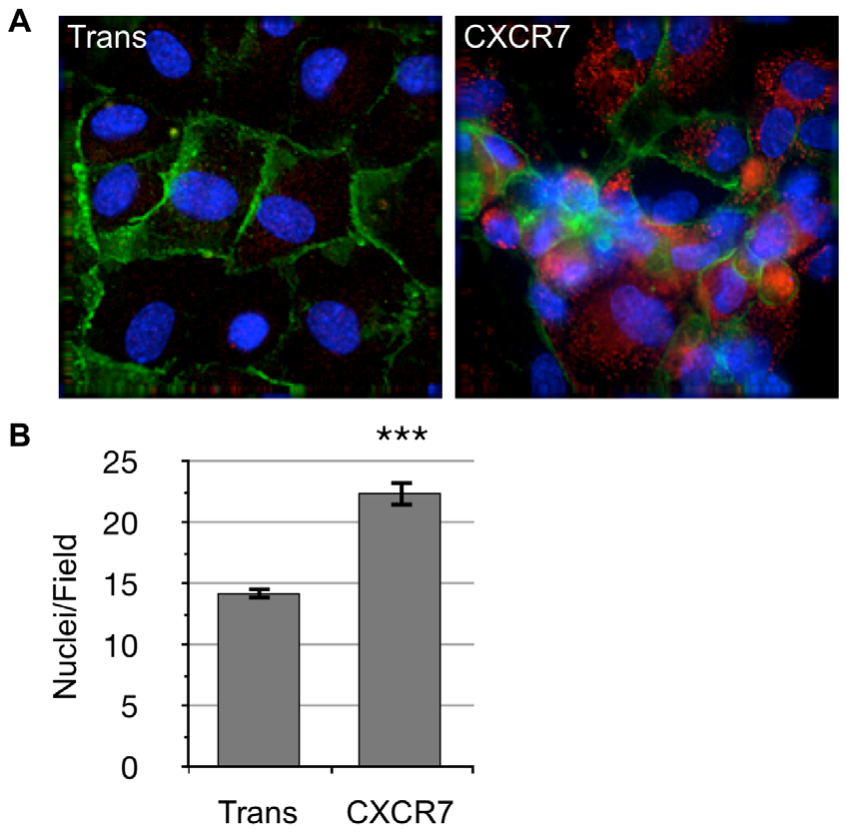
CXCR7+ EC are not contact inhibited. (A) pLEC were infected with Trans only or Trans+CXCR7. At 20 hours post-infection cells were trypsinized, counted and replated onto chamber slides at confluence and allowed to attach for a further 20 hours. Resulting monolayers were fixed and stained for DAPI (blue), CD31 (green), and CXCR7 (red). (B) Nuclei were counted in 10 random fields from 2 independent wells from 2 independent experiments (n = 40, *P*<0.0001).

In order to test whether post-confluent growth is associated with a loss of contact inhibition in CXCR7+ EC, we infected subconfluent pLEC with Trans only or Trans+CXCR7. At 20 hours post infection, cells were re-plated into smaller wells so that cells would form a confluent monolayer without needing to proliferate further. At 20 hours post-seeding (40 hours post-infection), wells were fixed and stained for CXCR7 and CD31/PECAM. Examination of re-plated cultures by deconvolution microscopy revealed a typical cobblestone monolayer formation with normal junctional CD31 staining in Trans controls. In contrast, CXCR7-transduced cultures formed highly abnormal monolayers with areas of multi-layered foci and low or absent CD31 junctional staining (Figure 2A). Comparing the number of nuclei per field in 40 random fields revealed that CXCR7+ cultures had significantly higher cell numbers per field, suggesting that CXCR7+ cultures continue to proliferate even when plated at confluence (Figure 2B). Taken together, these data reveal that CXCR7 expression allows EC to grow in a non-contact inhibited manner.

### CXCR7+ EC Display Altered Levels of Critical Proliferation Mediators

In order to identify potential mechanisms of CXCR7-mediated proliferation we first sought to determine whether CXCR7 expression affects the expression and/or phosphorylation of proteins involved in the regulation of cell division. We performed identical antibody microarrays (Full Moon Systems PEX100) using lysates from Trans or Trans+CXCR7 infected EC cultures and compared the two samples to identify proteins specifically altered in the presence of CXCR7. The complete list of altered expression and phosphorylation hits is available as supporting documentation (Table S1). Table 1 shows selected proteins altered by CXCR7 expression that are functionally associated with proliferation. Importantly, multiple antibody spots demonstrated downregulation of the key tumor suppressor Retinoblastoma protein (Rb) in CXCR7+ EC.

**Table 1:** Selected proliferation-related proteins displaying modified expression or phosphorylation in CXCR7+ EC.

Protein Name (antibody/phosphorylation site)	Expression Level (CXCR7/Trans)
GSK3a-b (Ab-216/279)	6.3
DAXX (Phospho-Ser668)	3.4
EGFR (Ab-1069)	2.2
p53 (Phospho-Thr81)	0.5
Stathmin 1(Ab-15)	0.5
p53 (Ab-18)	0.4
p53 (Ab-378)	0.4
Rb (Ab-780)	0.4
Rb (Ab-780)	0.4
Cyclin D3 (Ab-283)	0.4
Cyclin E1 (Phospho-Thr77))	0.4
Stathmin 1(Ab-37)	0.4
Rac1/cdc42 (Ab-71)	0.3

### Retinoblastoma Protein (Rb) Protein is Degraded in CXCR7+ EC

**Figure 3:**
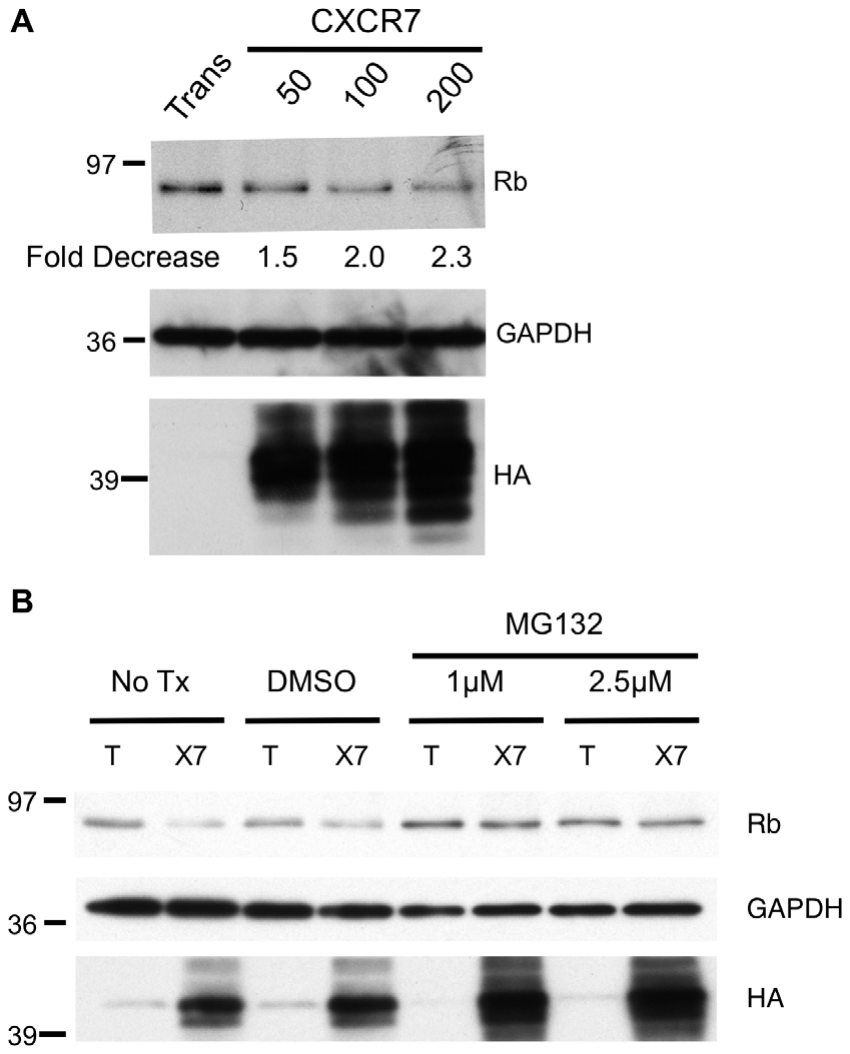
CXCR7 expression causes degradation of Rb in EC. (A) pLEC cultures were infected with Trans only (0) or Trans+CXCR7 at MOI 50, 100 or 200. At 20 hours post-infection, cells were lysed and analyzed by western blot for total Rb levels. Densitometry analysis was performed on Rb blots wherein each sample was normalized to GAPDH signal intensity and GAPDH-normalized fold decrease of Rb expression compared Trans is displayed beneath each dose (B) pLEC cultures were infected with Trans only or Trans+CXCR7. At 6 hours post-infection, media was replaced with media containing DMSO vehicle or media containing MG132 at 1 or 2.5 µM. At 20 hours post infection, cells were lysed and analyzed by western blot for total Rb levels.

The pocket protein family member Rb is a critical regulator of cell cycle progression. Aberrant inhibition of Rb function is a frequent occurrence in the course of human tumorigenesis [20]. Consistent with our microarray data, western blot analysis of lysates from pLEC cultures revealed a dose-dependent degradation of total Rb protein in CXCR7-expressing EC compared to Trans controls (Figure 3A). Densitometry analysis of nine sample sets from six independent experiments performed with CXCR7-transduction at an MOI of 200 revealed that this phenotype is reproducible with an average 2.8±0.3 fold decrease of total Rb protein in CXCR7+ cultures compared to Trans controls. Similar Rb degradation was observed in CXCR7-transduced pBEC (Figure S1B). Others have shown that Rb degradation can occur in cancer cells via a proteasome-mediated mechanism [21]. In order to test whether the proteasome is responsible for CXCR7-mediated Rb degradation, we performed similar experiments in which DMSO vehicle or the proteasome inhibitor MG132 was added to transduced cultures at 6 hours post-infection. Indeed, Rb expression is restored in CXCR7+ EC treated with MG132, demonstrating that Rb degradation in this system is mediated by the proteasome (Figure 3B). Taken together, these results demonstrate that CXCR7 expression in EC results in the proteasomal degradation of the tumor suppressor protein Rb, providing a potential mechanism for the aberrant proliferation seen in CXCR7+ EC cultures.

### Expression of CXCR7 Ligands and EC Growth Factors are not Significantly Altered in CXCR7+ EC

We next wanted to determine whether expression of the known CXCR7 chemokine ligands SDF-1/CXCL12 and ITAC/CXCL11 is altered in CXCR7-transduced EC cultures. In these experiments we transduced pLEC with increasing doses of CXCR7 adenovirus, harvested cells at 20 hours post-infection and performed quantitative RT-PCR analysis for mRNA expression of CXCR7, SDF-1/CXCL12 and ITAC/CXCL11 (Table 2). These results reveal that CXCR7 expression has no effect on expression of SDF-1/CXCL12 transcripts. We observed a small reproducible induction of ITAC/CXCL11 at higher doses of CXCR7. We wanted to confirm these results by determining the concentration of CXCR7 ligands in EC culture supernatants. Additionally, we wanted to rule out the possibility that the proliferation of CXCR7+ EC cultures was a result of indirect induction of secreted growth factors. For these experiments, we harvested supernatants at 20 hours post-infection from duplicate experiments in which pLEC were transduced with Trans or Trans+CXCR7. Cytokine levels in the supernatants were analyzed using a custom Quantibody® antibody array (Ray Biotech). We found that transduction of EC with CXCR7 does not significantly or reproducibly alter supernatant levels of any of the cytokines assayed (Table 3). We did detect low levels of ITAC/CXCL11 in some samples, consistent with our RT-PCR results. Although we were unable to detect SDF-1/CXCL12 in the culture supernatants, SDF-1/CXCL12 is known to be highly cell-associated through glycosaminoglycan interactions [22] and, as such, we cannot rule out the presence of SDF-1/CXCL12 in these cultures.

**Table 2:** Fold changes in mRNA expression in CXCR7-transduced EC compared to uninfected controls.

	CXCR7	SDF-1/CXCL12	ITAC/CXCL11
**Trans**	0.2±0.1	1.2±0.6	1.0±0.3
**CXCR7 MOI 50**	150.1±19.9	0.8±0.4	1.0±0
**CXCR7 MOI 100**	578.9±35	0.9±0.5	2.5±0.8
**CXCR7 MOI 200**	1114.9±117.4	1.0±0.5	2.8±0.1

Average ± standard error of two experimental replicates.

**Table 3:** Supernatant concentrations of selected cytokines in Trans or CXCR7-transduced EC.

	Experiment 1		Experiment 2			
	Trans	CXCR7	Trans	CXCR7	LOD	MAX
**SDF-1/CXCL12**	0.0	0.0	0.0	0.0	10.0	60,017
**I-TAC/CXCL11**	0.0	89.0	77.0	0.0	54.0	60,012
**6ckine/CCL21**	0.0	0.0	241.0	0.0	183.0	60,000
**ANG-1**	0.0	0.0	0.0	0.0	36.0	60,002
**ANG-2**	381471.0	465732.0	1447429.0	1162435.0	138.0	60,003
**EGF**	786496.0	820226.0	9955.0	9158.0	158.0	60,005
**ENA-78/CXCL5**	180.0	212.0	0.0	0.0	110.0	60,006
**HGF/SF**	301.0	0.0	502.0	0.0	199.0	60,008
**IL-8/CXCL8**	310714.0	374540.0	146329.0	125787.0	137.0	60,009
**IP-10/CXCL10**	0.0	0.0	0.0	0.0	28.0	60,011
**MIG/CXCL9**	644.0	790.0	3569.0	1844.0	282.0	60,014
**MIP-3b/CCL19**	0.0	0.0	0.0	0.0	28.0	60,015
**VEGF**	0.0	141.0	0.0	0.0	119.0	60,018
**VEGF-C**	0.0	0.0	0.0	0.0	81.0	60,020
**VEGF-D**	0.0	0.0	0.0	0.0	37.0	60,021

Quantities are displayed in pg/ml.

LOD: Limit of Detection; MAX: maximum concentration of standard curve.

ANG: angiopoietin; EGF: epidermal growth factor; HGF/SF: hepatocyte growth factor/scatter factor; VEGF: vascular endothelial growth factor.

### CXCR7-mediated Rb Degradation is Ligand-dependent but not Ligand-specific

**Figure 4:**
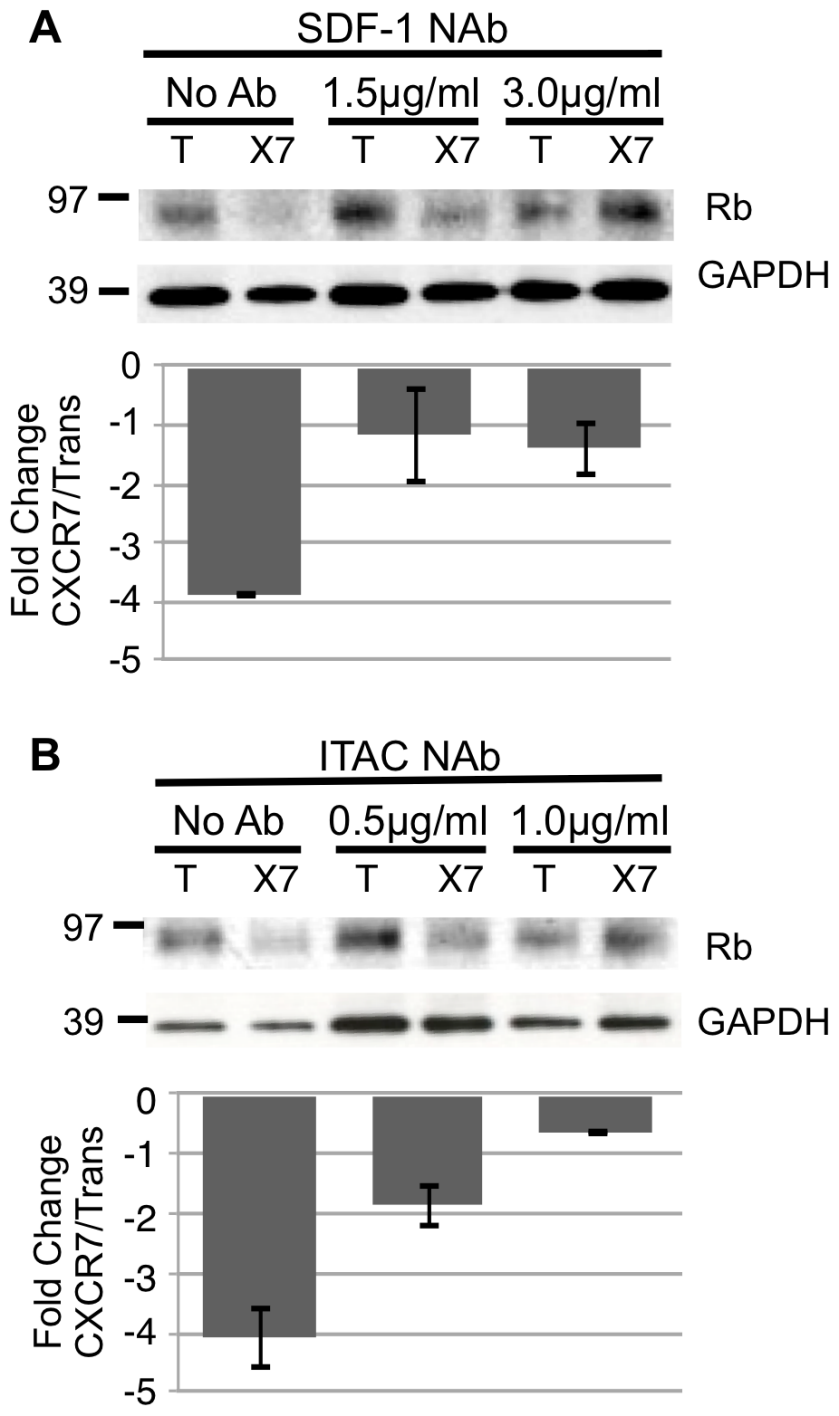
CXCR7-mediated Rb degradation requires ligand. pLEC cultures were infected with Trans only or Trans+CXCR7. At 6 hours post-infection, media was replaced with media only or media containing (A) SDF-1/CXCL12 neutralizing antibody at 1.5, or 3.0 µg/ml or (B) ITAC/CXCL11 neutralizing antibody at 0.5 or 1.0 µg/ml. At 20 hours post infection, cells were lysed and analyzed by western blot for total Rb levels. Densitometry analysis was performed and fold change values were calculated from GAPDH-normalized optical density ratios as described for Figure 3. Bar graphs (bottom) represent the average fold change at each condition for two independent experiments.

In order to determine whether Rb degradation in CXCR7+ EC is mediated by CXCR7 ligands, we performed experiments in which transduced pLEC cultures were treated with increasing doses of neutralizing antibodies directed against the two CXCR7 ligands SDF-1/CXCL12 or ITAC/CXCL11. Western blot analysis of cellular lysates revealed a dose-dependent rescue of Rb expression with neutralization of either SDF-1/CXCL12 (Figure 4A) or ITAC/CXCL11 (Figure 4B). The finding that SDF-1/CXCL12 neutralizing antibody abrogates CXCR7-mediated Rb degradation suggests that there is cell-associated SDF-1/CXCL12 present in pLEC cultures that was not detected in the supernatant. These results demonstrate that CXCR7-mediated Rb degradation requires CXCR7 ligand. Importantly, this phenotype is not ligand-specific because rescue of Rb can be achieved by neutralization of either CXCR7 ligand.

### CXCR7 Transduction does not Affect Surface Expression of CXCR3 and CXCR4

**Figure 5:**
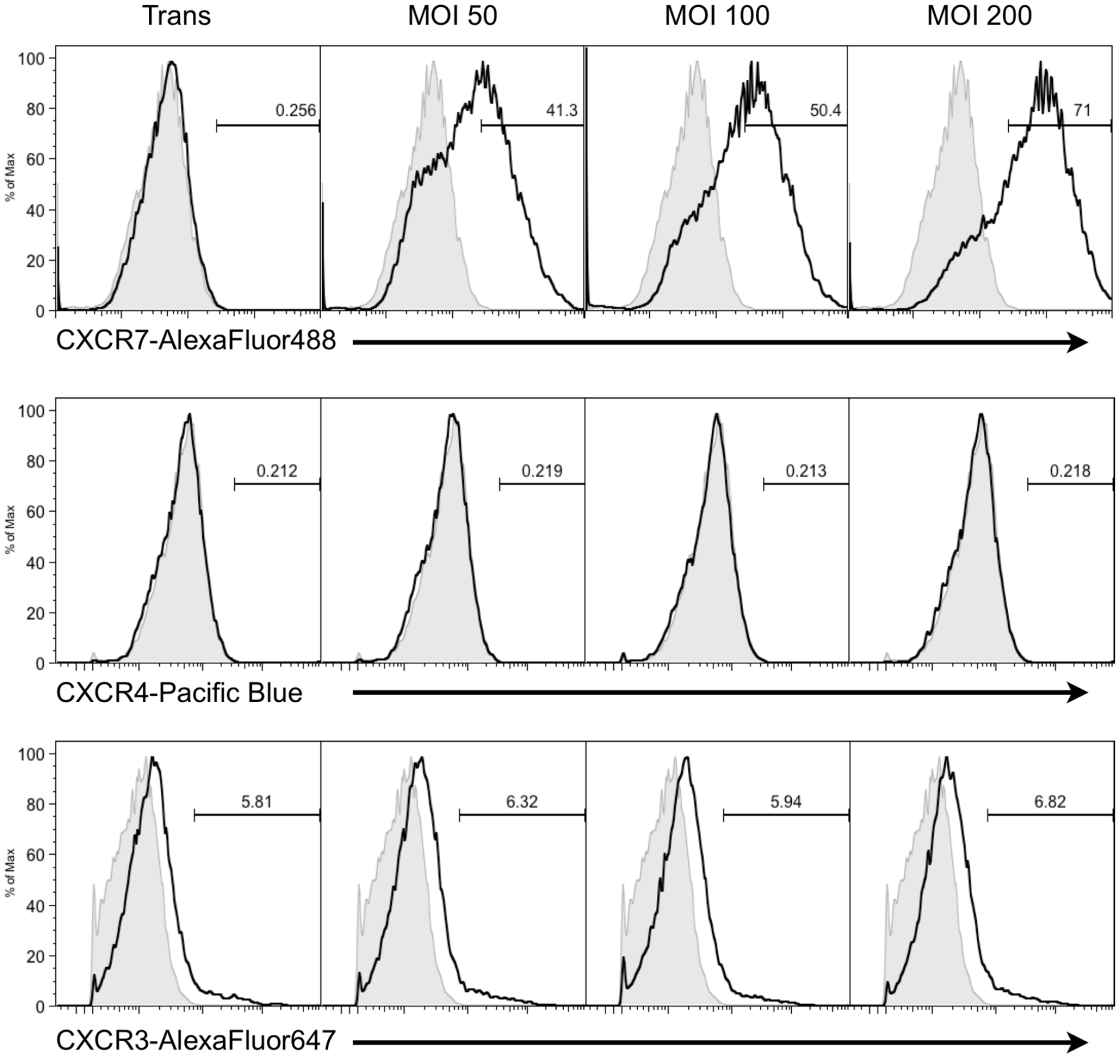
CXCR7 transduction does not affect surface expression of CXCR3 and CXCR4. pLEC cultures were infected with Trans only or Trans+CXCR7 at indicated doses. At 20 hours post-infection cells were harvested and stained by flow cytometry for CXCR7 via 8F11 monoclonal antibody, CXCR4 and CXCR3. Necrotic cells were excluded from the analysis via propidium iodide staining and scatter characteristics. Positive staining (black line) was compared to appropriate isotype control (grey fill) and positive populations were gated based on isotype control levels. Data is representative of two experimental replicates.

CXCR7 shares ligands with two other chemokine receptors; SDF-1/CXCL12 binds to both CXCR7 and CXCR4 while ITAC/CXCL11 binds to both CXCR7 and CXCR3 [9]. Indeed, it has been hypothesized that CXCR7 is not a functional chemokine receptor but a chemokine sink and that its primary function is to alter the availability of ligands for these alternate receptors [11], [23], [24]. This promiscuity makes receptor/ligand interactions difficult to dissect in EC, which can express both CXCR4 and CXCR3 [4], [25]. Therefore, we wanted to rule out participation of these alternate receptors in the ligand-dependent degradation of Rb seen in CXCR7+ cultures. We performed experiments in which we transduced pLEC with increasing concentrations of CXCR7 adenovirus and detected surface expression of CXCR7, CXCR3 and CXCR4 by flow cytometry (Figure 5). We observed dose-dependent increases in CXCR7 expression as expected. However, we observed no CXCR4 staining above isotype control in any condition and although a small number of CXCR3 positive EC were observed in these experiments (4% ±0.8 average from 8 samples in two experimental replicates), CXCR7 transduction did not alter CXCR3 levels. These results demonstrate that cell surface expression of alternate receptors for CXCR7 ligands in pLEC cultures is very low and is not affected by CXCR7 expression. Therefore, it is unlikely that the significant gain-of-function proliferation and Rb degradation phenotypes seen in CXCR7-transduced cultures is a result of signaling from these alternate receptors.

### Pharmacological Inhibition of CXCR7 Restores Rb Expression and Prevents Overconfluent Growth

**Figure 6:**
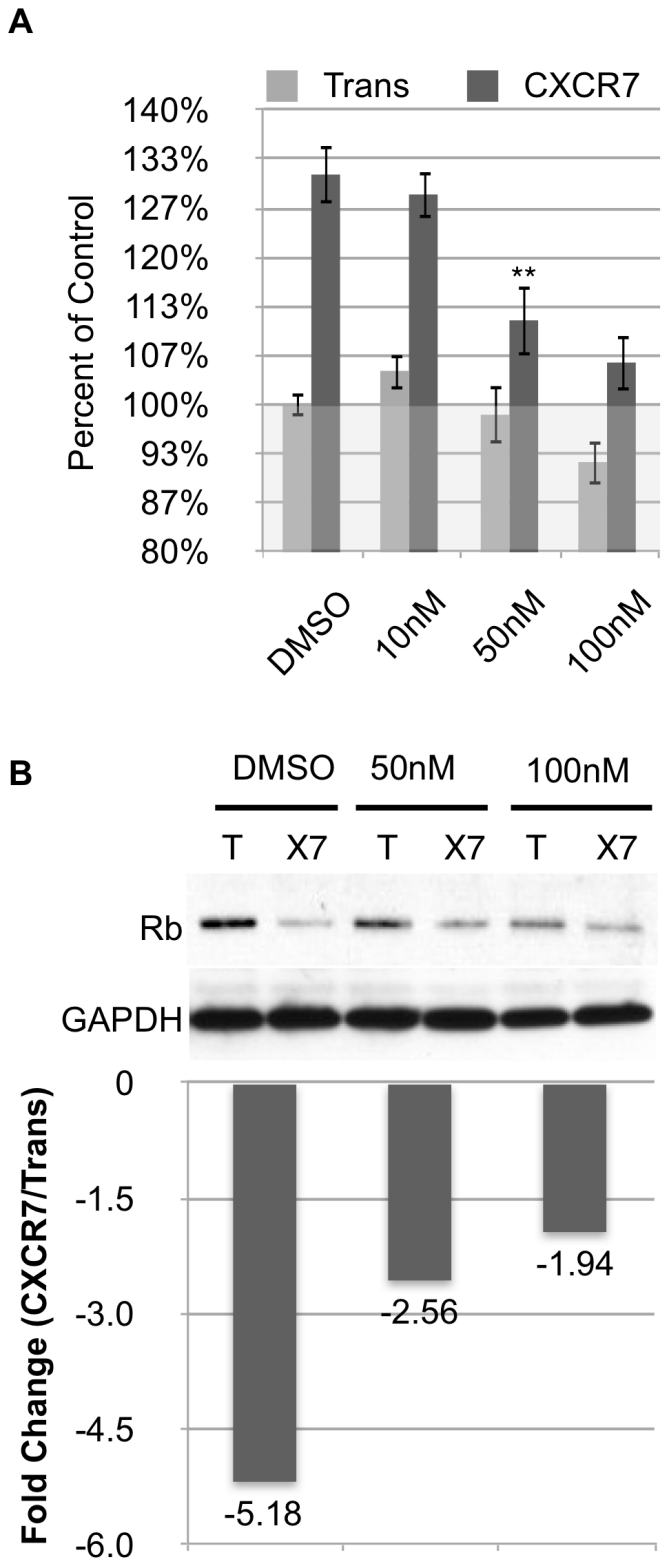
A small molecule inhibitor of CXCR7 prevents overconfluent growth and restores Rb expression. (A) Confluent pLEC cultures were infected with Trans only or Trans+CXCR7. At 6 hours post-infection, media was replaced with media containing DMSO vehicle control or CCX733 at 10 nM, 50 nM or 100 nM. At 20 hours post-infection, cells were lysed in the presence of Cyquant. Flourescence values are normalized to Trans+DMSO control. n>10 from two independent experiments. **P<0.01 (B) pLEC cultures were infected and treated as described for (A). At 20 hours post-infection, cells were lysed and analyzed by western blot for total Rb levels. Densitometry analysis was performed and fold change values were calculated from GAPDH-normalized optical density ratios as described for Figure 3.

In order to reinforce our hypothesis that CXCR7-mediated EC proliferation and Rb degradation is a result of CXCR7 signaling, we examined the effect of a small molecule inhibitor of CXCR7, CCX733, on proliferation and Rb protein levels in CXCR7+ EC. For these experiments, we transduced confluent pLEC cultures with Trans or Trans+CXCR7. At 6 hours post-infection, we added DMSO vehicle or increasing doses (10, 50 or 100 nM) of CCX733. While the inhibitor was ineffective at 10 nM, treatment with 50 nM CCX733 produced a statistically significant decrease in the overproliferation seen in CXCR7+ EC cultures. The effect was more pronounced at 100 nM, however we observed a low level of cytotoxicity at this dose indicated by a decrease in cell numbers in the control Trans cultures (Figure 6A). Similar experiments in which we harvested total cellular lysates and performed western blot analysis for Rb protein levels revealed a partial rescue of Rb levels in cultures treated with CCX733 (Figure 6B). Therefore, pharmacological inhibition of CXCR7 can prevent aberrant proliferation and Rb degradation in EC. Moreover, these results show a direct correlation between Rb levels and proliferative capacity in CXCR7+ EC.

### Expression of a CXCR7 Mutant Rescues Rb and Displays Lower Proliferation Levels

**Figure 7:**
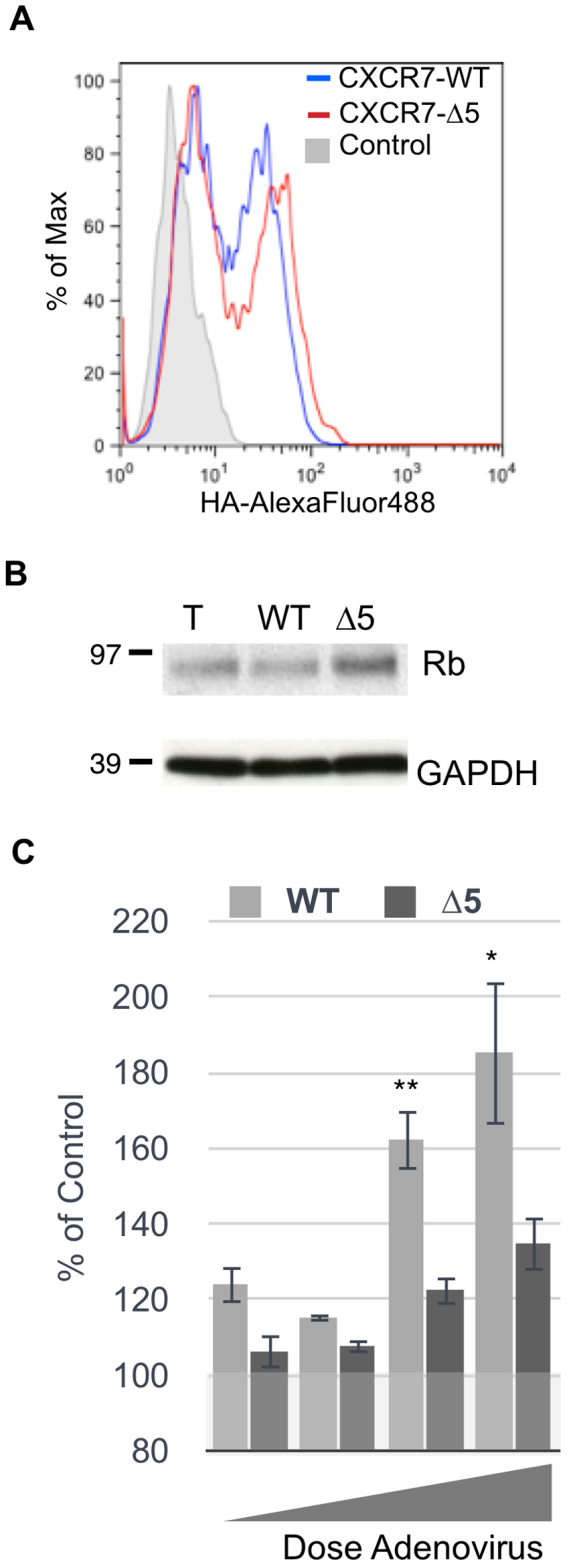
Expression of a CXCR7 mutant rescues Rb and prevents aberrant proliferation. (A) pLEC cultures were transduced with CXCR7 WT at MOI 200 or CXCR7-Δ5 at MOI 50. At 20 hours post-infection cells were stained for surface expression of the HA tag. Propidium Iodide was used to exclude necrotic cells. (B) Total lysates from pLEC cultures transduced as in (A) were harvested and analyzed by western blot analysis for total Rb protein. (C) Confluent pLEC cultures transduced with increasing doses of CXCR7-WT (MOI 50, 100, 200 or 300) or CXCR7-Δ5 (MOI 10, 20, 30 or 50). At 20 hours post-infection, cells were lysed in the presence of Cyquant. Fluorescence values are normalized to Trans only controls. n = 10. * *P* = 0.03, ** *P* = 0.001.

The N-terminal domain mediates high affinity chemokine interaction for both CXC [26] and CC [27] chemokine receptors. Therefore, we deleted five N-terminal amino acids from the CXCR7 sequence in our existing CXCR7 adenovirus vector (CXCR7-Δ5). The N-terminal HA-tag was retained to aid in detection. Flow cytometry analysis of pLEC transduced with CXCR7-Δ5 demonstrated that the modified protein is expressed on the cell surface (Figure 7A). In contrast to wild-type (WT) CXCR7 transduction, lysates from CXCR7-Δ5-transduced cultures displayed no degradation of Rb protein by western blot analysis (Figure 7B). Moreover, CyQuant analysis of confluent pLEC cultures transduced with increasing levels of CXCR7-Δ5 display decreased proliferation compared to CXCR7-WT (Figure 7C). Taken together, these results indicate that the proliferation phenotype associated with CXCR7-expression in EC can be abrogated by expression of a CXCR7 mutant, supporting our hypothesis that these phenotypes require interaction between CXCR7 and its chemokine ligands and further demonstrating that the degradation of Rb corresponds to proliferative capacity in these cultures.

## Discussion

Since its identification as an alternate SDF-1/CXCL12 binding receptor, CXCR7 has been the subject of intense study in the cancer field. However, dissection of CXCR7 signaling and function has proven difficult for several reasons. First and most importantly, the promiscuity of CXCR7 ligands and the widespread expression of CXCR4 and CXCR3 in tumor tissues [6] makes the dissection of CXCR7-specific signaling particularly challenging. Moreover, tumor cells can produce both SDF-1/CXCL12 [5] and ITAC/CXCL11 [28], [29], creating a complex signaling background and making experiments with exogenous chemokine ligand difficult to interpret. Third, it is clear from the current lack of consensus in the CXCR7 literature that CXCR7 signaling is likely to be both cell-type specific and ligand-specific, a phenomenon that has been described for other G-protein coupled receptors (GPCRs) [30] and chemokine receptors [31]. The latter challenge, in particular, necessitates careful examination of CXCR7 function in the cell type of interest, because the biological effects of CXCR7 expression and signaling are not necessarily universal between tissue types.

To our knowledge, all of the studies examining CXCR7 expression in tumor vasculature have used CD31/PECAM-1 as a marker of EC. As such, the vessels represented could be blood vascular, lymphatics or, most likely, both. Although we have performed the majority of experiments in the current study using primary lymphatic EC, we have shown that aberrant proliferation and Rb degradation occurs in blood vascular lineage EC as well (Figure S1). As such, the physiological implications of the current study are generalizable to both the lymphatic and blood vascular circulatory systems. The importance of tumor lymphatic vasculature to tumor immune surveillance [32] as well as the metastatic spread of tumors to the lymph nodes [33], [34] is well established. We believe that our current data supports further study into the contribution of CXCR7 expression in tumor-associated lymphatic vessels to tumor growth and metastasis.

Pathologically-induced CXCR7 has recently been shown to drive proliferation of pulmonary microvascular EC in models of pulmonary hypertension *in vitro* [35] and *in vivo* [19]. Moreover, the latter study demonstrated that pharmacological inhibition of CXCR7 could prevent vascular proliferation caused by chronic hypoxia-induced pulmonary hypertension. While these results are not necessarily translatable to tumor vascular dysfunction we believe that, when taken together with our current study in primary human EC and the wealth of histopathological data showing that CXCR7 is highly expressed in tumor vasculature, the potential of CXCR7 as an antiangiogenic target deserves further study.

When CXCR7 was still the orphan GPCR known as RDC1, our laboratory demonstrated that CXCR7 is highly upregulated in EC infected with Kaposi Sarcoma Herpesvirus (KSHV) [36]. KSHV is the etiological agent of Kaposi Sarcoma (KS) an angioproliferative tumor of the viscera, skin and mucosa in which the tumor cell is of endothelial origin [37]. Importantly, in our original study, knockdown of CXCR7 by RNA interference abrogated the ability of KSHV to produce a transformed phenotype in EC cultures, providing the first evidence that aberrant expression of CXCR7 contributes to malignancy. To our knowledge, KS is the only neoplasm studied to date that displays uniformly high levels of CXCR7 on tumor cells, and we have observed that levels of CXCR7 expression correlate with disease progression (Moses et. al, unpublished observations). Results from our current study provide CXCR7-mediated proliferation as a potential mechanism for the growth of KS tumors and establish CXCR7 as a potential target for the treatment of KS. Indeed, the expression of CXCR7 in other angioproliferative neoplasms will be of great interest in future studies.

There is significant evidence in the literature that CXCR7 alters the function of CXCR4 either by direct interaction or manipulation of ligand availability [23], [24], [38]. Experiments employing CXCR4 inhibitors would further strengthen the conclusion that CXCR4 is not involved in the observed phenotypes. Unfortunately, two CXCR4 antagonists AMD3100 and TC14012 are known agonists for CXCR7 [39], [40] and, to our knowledge, the other CXCR4 antagonists have not been tested for CXCR7 binding capacity. Therefore, we believe that the use of CXCR4 inhibitors would be uninterpretable in our EC system. We have demonstrated that surface expression of both CXCR3 and CXCR4 are very low in these cultures and are not altered by CXCR7 expression (Figure 5), and we have utilized both a small molecule inhibitor of CXCR7 and targeted mutagenesis of the CXCR7 N-terminus to abrogate the observed phenotypes (Figures 6 & 7). We believe that, taken together, the data presented in the current study supports the conclusion that the proliferative phenotypes observed in CXCR7+ EC are a direct result of CXCR7 signaling and represent the most rigorous demonstration thereof that is possible given the complexity of the system and the tools currently available.

In the current study, we demonstrate that CXCR7 expression causes the proteasome-mediated degradation of Rb protein in EC, providing a specific cellular mechanism for CXCR7-mediated proliferation. To our knowledge, this is the first demonstration of CXCR7-mediated inhibition of a tumor suppressor pathway. The Rb regulatory pathway is thought to be dysfunctional in most human cancer [20]. Indeed, the function of Rb as a master regulator of cellular proliferation was first appreciated when it was shown to be the target of several viral oncogenes. Virus-mediated inhibition of Rb function proceeds by a variety of mechanisms including (1) direct phosphorylation of Rb, (2) steric inhibition of Rb interactions with the E2F transcription factor and (3) targeting of Rb protein for degradation [41], [42]. These virus-mediated oncogenic mechanisms provide clues to potential cellular mechanisms of Rb inactivation in uninfected malignant cells, some of which have since been revealed. Overexpression of the E3 ubiquitin ligase MDM2 is one of the only cellular mechanisms for degradation of Rb in cancer cells that has been characterized to date [21], but the factors driving MDM2 overexpression in cancer systems remain obscure. It will be interesting to determine whether aberrant expression of CXCR7 in EC results in overexpression or activation of MDM2 and whether MDM2 affects Rb expression in EC. Moreover, if the phenotype we demonstrate is common and CXCR7 signaling causes degradation of Rb in tumor cells in other cancer systems, the inhibition of CXCR7 could be a powerful target for the treatment of malignant cellular proliferation in general.

Although we have chosen to focus on Rb as a protein of interest from our antibody microarray screen of CXCR7-modulated proteins, we provide the complete list of proteins and phosphorylation sites found to be modified by CXCR7 expression as supporting information (Table S1). In addition to identifying additional proliferation-modulators affected by CXCR7 - including, notably, the tumor suppressor protein p53 (Table 1) – these data also suggest that CXCR7 may participate in the control of apoptosis and participate in the response of EC to environmental stress. We anticipate that this data set will help further the studies of researchers in multiple fields related to EC biology and CXCR7 signaling.

## Supporting Information

Figure S1 **CXCR7 expression in pBEC results in post-confluent proliferation and causes Rb degradation.** Confluent pBEC cultures were infected with Trans only or Trans+CXCR7 at MOI 100. At 20 hours post-infection cells were (A) lysed in the presence of CyQuant or (B) lysed and analyzed by western blot for total Rb levels. Fluorescence values are normalized to uninfected controls. n = 18 from three independent experiments. *** *P*<0.001.
(PDF)

Table S1 (PDF)
