# Supplementary figures and images for: Aberrant Proliferation in CXCR7+ Endothelial Cells via Degradation of the Retinoblastoma Protein
Source: PLoS One. 2013 Jul 23;8(7):e69828. doi: 10.1371/journal.pone.0069828 (PMC3720914; doi:10.1371/journal.pone.0069828)

Supporting Figure 1

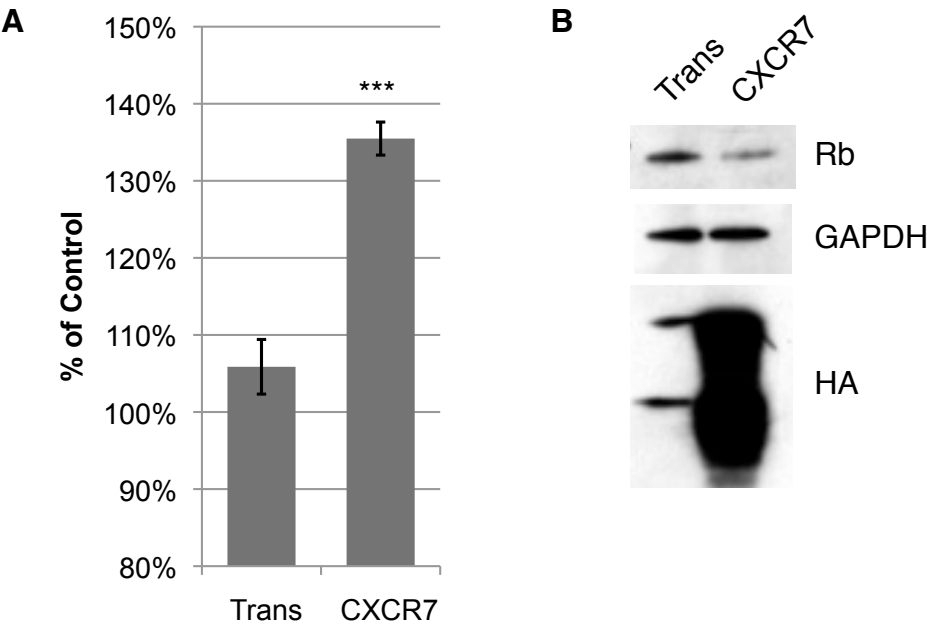

Supplement: Figure S1 — CXCR7 expression in pBEC results in post-confluent proliferation and causes Rb degradation. Confluent pBEC cultures were infected with Trans only or Trans+CXCR7 at MOI 100. At 20 hours post-infection cells were (A) lysed in the presence of CyQuant or (B) lysed and analyzed by western blot for total Rb levels. Fluorescence values are normalized to uninfected controls. n = 18 from three independent experiments. *** P<0.001. (PDF) [file pone.0069828.s001.pdf]
