# Supplementary material for: Aberrant Proliferation in CXCR7+ Endothelial Cells via Degradation of the Retinoblastoma Protein
Source: PLoS One. 2013 Jul 23;8(7):e69828. doi: 10.1371/journal.pone.0069828 (PMC3720914; doi:10.1371/journal.pone.0069828)
Supplement: Table S1 — (PDF) [file pone.0069828.s002.pdf]

Table S1A - Proteins/Phosphorylation Sites upregulated >1.5 fold in CXCR7+ EC

**Fold CXCR7/ Trans**	**PEX100 Reference**	**Description**
22.05	292	BCL-2 (Ab-69)
9.30	439	PKD2 (Ab-876)
6.32	427	GSK3a-b (Ab-216/279)
3.37	1232	DAXX (Phospho-Ser668)
3.31	1017	Tyrosine Hydroxylase (Phospho-Ser19)
3.19	231	FOXO1A/3A (Phospho-Ser322/325)
3.16	228	FAK (Phospho-Tyr407)
2.87	248	GTPase activating protein (Phopspho-Ser387)
2.40	13	NFkB-p65 (Phospho-Ser468)
2.20	1095	FRS2 (Phospho-Tyr436)
2.16	622	EGFR (Ab-1069)
2.16	291	BAX (Ab-167)
2.02	751	ATPase (Phospho-Ser16)
1.87	34	Histone H3.1 (Phospho-Ser10)
1.86	254	RapGEF1 (Phospho-Tyr504)
1.85	192	GATA1 (Ab-142)
1.76	717	Opioid Receptor (Ab-375)
1.68	748	ATF-1 (Phospho-Ser63)
1.64	733	PKC delta (Phospho-Thr505)
1.60	1206	BCR (Ab-177)
1.60	1036	5.25
1.59	1169	Zap-70 (Phospho-Tyr319)
1.59	11	NFkB-p65 (Phospho-Ser276)
1.55	1025	HSP27 (Ab-15)
1.54	351	Estrogen Receptor-α (Ab-118)

Table S1B - Proteins/Phosphorylation Sites downregulated >0.5 fold in CXCR7+ EC

Fold CXCR7/ Trans	PEX100 Reference	Description
0.50	728	p53 (Phospho-Thr81)
0.50	1192	NFkB-p100/p52 (Ab-865)
0.50	910	MYPT1 (Phospho-Thr696)
0.50	805	MAP3K7/TAK1 (Ab-187)
0.50	864	LIMK1 (Ab-508)
0.50	842	DARPP-32 (Phospho-Thr75)
0.50	969	AurA (Ab-342)
0.50	702	14-3-3 zeta (Ab-58)
0.49	414	MSK1 (Phospho-Ser212)
0.49	1042	IRS-1 (Ab-312)
0.49	982	IL3R (Ab-593)
0.49	190	MEF2A (Ab-312)
0.49	954	IGFBP-3 (Ab-183)
0.49	467	LYN (Ab-507)
0.49	755	FGFR1 (Phospho-Tyr654)
0.49	540	BIM (Ab-69/65)
0.49	1274	RyR2 (Ab-2808)
0.49	925	EPHA2/3/4 (Phospho-Tyr588/596)
0.49	987	Chk1 (Phospho-Ser345)
0.49	1203	IkB-alpha (Ab-42)
0.49	665	Dok-1 (Phospho-Tyr398)
0.49	317	STAT6 (Phospho-Thr645)
0.49	1284	Zap-70 (Ab-292)
0.49	352	Estrogen Receptor-α (Ab-167)
0.49	425	Catenin beta (CTNNB) (Ab-33)
0.49	73	Stathmin 1(Ab-15)
0.49	301	PKC epsilon (Ab-729)

Fold CXCR7/ Trans	PEX100 Reference	Description
0.49	922	p130Cas (Phospho-Tyr165)
0.49	1149	CD5 (Ab-453)
0.49	760	Abl1 (Ab-204)
0.49	802	CK1-A (Ab-321)
0.48	872	SHP-2 (Ab-542)
0.48	1081	Interferon-gamma receptor alpha chain precursor (Phospho-Tyr457)
0.48	828	Tau (Phospho-Ser404)
0.48	1096	Ephrin B1/B2/B3 (Phospho-Tyr324)
0.48	40	c-Jun (Ab-93)
0.48	787	Smad2 (Ab-255)
0.48	897	BCL-2 (Phospho-Thr69)
0.48	838	14-3-3 zeta (Phospho-Ser58)
0.48	938	FAK (Ab-407)
0.48	38	Rel (Ab-503)
0.48	806	MAP3K7/TAK1 (Ab-439)
0.48	895	A-RAF (Phospho-Tyr301/302)
0.48	390	Raf1 (Phospho-Tyr341)
0.48	1050	P70S6k (Phospho-Thr421)
0.48	847	IkB-beta (Phospho-Ser23)
0.47	1010	SHP-2 (Phospho-Tyr542)
0.47	889	EGFR (Phospho-Thr693)
0.47	783	HER3/ErbB3 (Ab-1222)
0.47	491	Estrogen Receptor-α (Phospho-Ser167)
0.47	471	ATPase (Ab-16)
0.47	1016	Trk B (Phospho-Tyr515)
0.47	69	IRS-1 (Ab-636)
0.47	934	CD3Z (Ab-142)

Fold CXCR7/ Trans	PEX100 Reference	Description
0.47	93	FAK (Phospho-Ser910)
0.47	9	PTEN (Phospho-Ser380)
0.47	267	HRS (Phospho-Tyr334)
0.47	1176	eEF2K (Phospho-Ser366)
0.47	620	DNA-PK (Ab-2638)
0.47	817	p53 (Phospho-Ser37)
0.46	700	Caveolin-1 (Ab-14)
0.46	697	Rb (Ab-807)
0.46	685	Tau (Ab-205)
0.46	88	Arrestin-1 (Phospho-Ser412)
0.46	955	MKP-1 (Ab-359)
0.46	857	Chk2 (Ab-516)
0.46	1008	Shc (Phospho-Tyr427)
0.46	484	BCL-XL (Phospho-Ser62)
0.46	309	HNF4α (Phospho-Ser304)
0.46	1199	VASP (Ab-238)
0.46	830	Catenin beta (Phospho-Thr41/Ser45)
0.46	800	SEK1/MKK4/JNKK1 (Ab-257)
0.46	703	ADD1 (Ab-726)
0.46	555	AKT1 (Phospho-Tyr474)
0.45	1092	CK1-A/A2 (Phospho-Tyr294)
0.45	1193	NFkB-p100/p52 (Ab-869)
0.45	953	IGF2R (Ab-2409)
0.45	593	AKT1 (Ab-308)
0.45	907	PKC epsilon (Phospho-Ser729)
0.45	952	Hsp90 co-chaperone Cdc37 (Ab-13)
0.45	1049	SYK (Phospho-Tyr348)

Fold CXCR7/ Trans	PEX100 Reference	Description
0.45	785	HER4/ErbB4 (Ab-1284)
0.45	372	Cortactin (Ab-421)
0.45	865	SEK1/MKK4 (Ab-261)
0.45	387	PEA-15 (Phospho-Ser116)
0.45	33	p27Kip1 (Phospho-Thr187)
0.44	945	LAT (Ab-191)
0.44	461	Tuberin (Ab-981)
0.44	698	Rb (Ab-780)
0.44	1001	Chk1 (Phospho-Ser280)
0.44	845	GABA-RB (Phospho-Ser434)
0.44	357	EGFR (Ab-1070)
0.44	867	Histone H3.1 (Ab-10)
0.44	1119	14-3-3 zeta/beta (Ab-184/186)
0.44	1201	Zap-70 (Ab-493)
0.44	362	GluR1 (Ab-849)
0.44	61	4E-BP1 (Ab-36)
0.43	946	LCK (Ab-504)
0.43	1124	Ezrin (Ab-478)
0.43	585	Gab2 (Phospho-Tyr643)
0.43	1028	ATM (Ab-1981)
0.43	873	SHP-2 (Ab-580)
0.43	16	NFkB-p100/p52 (Phospho-Ser869)
0.43	1204	STAT1 (Ab-727)
0.43	874	Synaptotagmin (Ab-309)
0.43	1298	Raf1 (Ab-289)
0.43	692	Androgen Receptor (Ab-213)
0.42	64	Stathmin 1(Ab-37)

Fold CXCR7/ Trans	PEX100 Reference	Description
0.42	1187	NFkB-p65 (Ab-254)
0.42	1132	Abl1 (Ab-754/735)
0.42	1190	NFkB-p65 (Ab-468)
0.42	962	IKK gamma (Ab-85)
0.42	1182	PDK1 (Ab-241)
0.42	209	FGFR1 (Ab-154)
0.42	984	IR (Ab-1361)
0.42	543	Gab1 (Ab-627)
0.42	1226	AKT1 (Phospho-Thr450)
0.42	516	IGF-1R (Ab-1161)
0.42	1126	NFkB-p65 (Ab-281)
0.42	1315	STAM2 (Ab-192)
0.42	971	AurB/C (Ab-202/175)
0.42	67	EGFR (Ab-1197)
0.42	391	Raf1(Phospho-Ser621)
0.41	861	JAK2 (Ab-1007)
0.41	1222	4E-BP1 (Phospho-Thr70)
0.41	189	Elk1 (Ab-417)
0.41	786	Smad1 (Ab-187)
0.41	1013	Synaptotagmin (Phospho-Ser309)
0.41	885	Filamin A (Ab-2152)
0.41	515	VEGFR2 (Ab-951)
0.41	1196	Src (Ab-529)
0.41	712	IkB-epsilon (Ab-22)
0.41	532	eIF2A (Ab-51)
0.40	832	CDC25C (Phospho-Ser216)
0.40	981	TOP2A/DNA topoisomerase II (Ab-1106)
0.40	792	Smad3 (Ab-179)

Fold CXCR7/ Trans	PEX100 Reference	Description
0.40	659	Src (Phospho-Tyr418)
0.40	759	4E-BP1 (Ab-70)
0.40	169	VASP (Phospho-Ser157)
0.39	349	Estrogen Receptor-α (Ab-104)
0.39	522	p53 (Ab-18)
0.39	1277	Tuberin/TSC2 (Ab-939)
0.39	738	Ras-GRF1 (Phospho-Ser916)
0.39	1068	BAD (Phospho-Ser91/128)
0.39	600	Pyk2 (Ab-881)
0.39	767	Cyclin D3 (Ab-283)
0.39	1221	4E-BP1 (Phospho-Ser65)
0.39	283	PLC beta3 (Ab-537)
0.39	1029	FAK (Ab-925)
0.39	911	MYPT1 (Phospho-Thr-853)
0.38	529	Tau (Ab-396)
0.38	932	Arrestin-1 (Ab-412)
0.38	849	Keratin 18 (Phospho-Ser33)
0.38	776	IKK-a/b (Ab-176)
0.38	563	Cyclin E1 (Phospho-Thr77))
0.38	1316	FER (Ab-402)
0.38	854	Chk1 (Ab-317)
0.37	1194	NFkB-p105/p50 (Ab-337)
0.37	1318	claudin 7 (Ab-210)
0.37	1112	P90RSK (Ab-573)
0.37	921	Myosin regulatory light chain 2 (Phospho-Ser18)
0.37	920	Mst1/Mst2 (Phospho-Thr183)
0.37	1129	p53 (Ab-378)

Fold CXCR7/ Trans	PEX100 Reference	Description
0.37	690	G3BP-1 (Ab-232)
0.36	778	FosB (Ab-27)
0.36	991	LIMK1 (Phospho-Thr508)
0.36	592	Smad3 (Ab-425)
0.36	939	FOXO1/3/4-PAN (Ab-24/32)
0.36	504	Histone H2A.X (Phospho-Ser139)
0.36	618	c-Raf (Ab-43)
0.36	411	MKK3/MAP2K3 (Phospho-Thr222)
0.35	415	MSK2 (Phospho-Thr568)
0.35	1208	Paxillin (Ab-31)
0.35	135	IRS-1 (Ab-794)
0.35	1120	Catenin beta (CTNNB) (Ab-489)
0.35	1280	VAV1 (Ab-160)
0.34	368	Synapsin (Ab-9)
0.34	1011	SHP-2 (Phospho-Tyr580)
0.34	774	IkB-beta (Ab-19)
0.34	637	p130Cas (Ab-165)
0.33	1220	SYK (Phospho-Tyr525)
0.33	1210	Rac1/cdc42 (Ab-71)
0.33	902	GAB1 (Phospho-Tyr659)
0.33	195	STAT1 (Ab-701)
0.33	1012	Smad3 (Phospho-Ser425)
0.32	710	GABA-RB (Ab-434)
0.32	742	SREBP-1 (Phospho-Ser439)
0.32	711	HSP90B (Ab-254)
0.31	536	Ret (Ab-905)
0.31	891	14-3-3 theta/tau (Phospho-Ser232)

Fold CXCR7/ Trans	PEX100 Reference	Description
0.31	927	Ephrin-B1 (Phospho-Tyr317)
0.31	1186	PTEN (Ab-380)
0.31	1087	PIP5K (Phospho-Ser307)
0.30	249	Hsp90 co-chaperone Cdc37 (Phospho-Ser13)
0.30	285	14-3-3 theta/tau (Ab-232)
0.30	719	Shc (Ab-349)
0.30	1207	MSK1 (Ab-376)
0.30	866	ASK1 (Ab-966)
0.29	373	Cortactin (Ab-466)
0.29	441	Rb-like-2 (RBL2) (Ab-952)
0.29	1163	JAK2 (Phospho-Tyr1007)
0.29	951	GTPase activating protein (Ab-387)
0.29	940	FOXO1A (Ab-329)
0.29	730	PAK2 (Phospho-Ser20)
0.29	1075	Caspase 9 (Phospho-Ser196)
0.29	586	MAPKAPK2 (Phospho-Ser272)
0.29	511	P73 (Ab-99)
0.29	894	ACTIN Pan(a/b/g) (Phospho-Tyr55/53)
0.28	1164	IkB-alpha (Phospho-Ser32/36)
0.28	789	Smad2 (ab-245)
0.28	1009	SHP-1 (Phospho-Tyr536)
0.28	424	P38MAPK (Ab-180)
0.27	835	NMDAR1 (Phospho-Ser897)
0.26	853	Chk1 (Ab-280)
0.26	975	Interferon-gamma receptor alpha chain precursor (Ab-457)
0.26	777	FAS (Ab-291)
0.26	1027	Chk1 (Ab-345)

Fold CXCR7/ Trans	PEX100 Reference	Description
0.26	1303	LCK (Ab-59)
0.25	963	mTOR (Ab-2446)
0.25	950	GRB10/Growth factor receptor-bound protein 10 (Ab-67)
0.25	1244	Cytokeratin 8 (Phospho-Ser431)
0.24	635	Mst1/Mst2 (Ab-183)
0.24	779	GRK1 (Ab-21)
0.22	875	Synaptotagmin (Ab-202)
0.22	790	Smad2 (Ab-220)
0.21	1031	p27Kip1 (Ab-10)
0.21	448	Caspase 9 (Ab-153)
0.21	632	WAVE1 (Ab-125)
0.19	1047	Pyk2 (Phospho-Tyr580)
0.17	1283	WASP (Ab-290)
0.16	704	AMPK1 (Ab-174)
0.14	914	PPAR-b (Phospho-Thr1457)
0.14	541	CPI17α (Ab-38)
0.14	968	AurB (Ab-232)
0.14	912	PLCG1 (Phospho-Tyr1253)
0.13	803	HSP 90-beta (Ab-226)
0.13	1291	P70S6K (Ab-427)
0.12	615	Cytokeratin 18 (Ab-52)
0.12	788	Smad2 (Ab-250)
0.12	1080	CD28 (Phospho-Tyr218)
0.10	949	CASP2 (Ab-157)
0.07	965	Rho/Rac guanine nucleotide exchange factor 2 (Ab-885)
0.07	416	VE-Cadherin (Phospho-Tyr731)
0.05	893	6-phosphofructo-2-kinase/fructose-2,6-biphosphatase 2 (PFKFB2) (Phospho-Ser483)
0.04	609	CDC25B (Ab-353)
